# Physiological responses to water deficiency in bread wheat (Triticum aestivum L.) lines with genetically different leaf pubescence

**DOI:** 10.18699/VJ20.678

**Published:** 2020-12

**Authors:** S.V. Osipova, A.V. Rudikovskii, A.V. Permyakov, E.G. Rudikovskaya, M.D. Permyakova, V.V. Verkhoturov, T.A. Pshenichnikova

**Affiliations:** Siberian Institute of Plant Physiology and Biochemistry of Siberian Branch of the Russian Academy of Sciences, Irkutsk, Russia Irkutsk State University, Irkutsk, Russia; Siberian Institute of Plant Physiology and Biochemistry of Siberian Branch of the Russian Academy of Sciences, Irkutsk, Russia; Siberian Institute of Plant Physiology and Biochemistry of Siberian Branch of the Russian Academy of Sciences, Irkutsk, Russia; Siberian Institute of Plant Physiology and Biochemistry of Siberian Branch of the Russian Academy of Sciences, Irkutsk, Russia; Siberian Institute of Plant Physiology and Biochemistry of Siberian Branch of the Russian Academy of Sciences, Irkutsk, Russia; National Research Irkutsk State Technical University, Irkutsk, Russia; Institute of Cytology and Genetics of Siberian Branch of the Russian Academy of Sciences, Novosibirsk, Russia

**Keywords:** drought tolerance, leaf pubescent genes;, isogenic lines, Triticum aestivum L., chlorophyll fluorescence, ascorbate-glutathione cycle enzymes, productivity, засухоустойчивость, гены опушения листа, изогенные линии, Triticum aestivum L., флуоресценция хлорофилла, ферменты аскорбат-глутатионового цикла, продуктивность

## Abstract

Studying the relationship between leaf pubescence and drought resistance is important for assessing Triticum aestivum L. genetic resources. The aim of the work was to assess resistance of common wheat genotypes with different composition and allelic state of genes that determine the leaf pubescence phenotype. We compared the drought resistance wheat variety Saratovskaya 29 (S29) with densely pubescent leaves, carrying the dominant alleles of the Hl1 and Hl3 genes, and two near isogenic lines, i: S29 hl1, hl3 and i: S29 Hl2aesp, with the introgression of the additional pubescence gene from diploid species Aegilops speltoides. Under controlled conditions of the climatic chamber, the photosynthetic pigments content, the activity of ascorbate-glutathione cycle enzymes and also the parameters of chlorophyll fluorescence used to assess the physiological state of the plants photosynthetic apparatus were studied in the leaves of S29 and the lines. Tolerance was evaluated using the comprehensive index D, calculated on the basis of the studied physiological characteristics. The recessive state of pubescence genes, as well as the introduction of the additional Hl2aesp gene, led to a 6-fold decrease in D. Under the water deficit influence, the fluorescence parameters profile changed in the lines, and the viability index decreased compared with S29. Under drought, the activity of ascorbate peroxidase, glutathione reductase and dehydroascorbate reductase in the line i: S29 hl1, hl3 decreased 1.9, 3.3 and 2.3 times, in the line i: S29 Hl2aesp it decreased 1.8, 3.6 and 1.8 times respectively, compared with S29. In a hydroponic greenhouse, line productivity was studied. Compared with S29, the thousand grains mass in the line i: S29 hl1, hl3 under water deficit was reduced. The productivity of the line i: S29 Hl2aesp was significantly reduced regardless of water supply conditions in comparison with S29. Presumably, the revealed effects are associated with violations of cross-regulatory interactions between the proteins of the trichome formation network and transcription factors that regulate plant growth and stress response.

## Abstract (ru)

Изучение взаимосвязи опушения листьев и засухоустойчивости важно для оценки генетических ресурсов Triticum aestivum L. Целью работы был анализ устойчивости к дефициту воды генотипов мягкой пшеницы с различными составом и аллельным состоянием генов, определяющих фенотип опушения листьев. Мы сравнили засухоустойчивый сорт пшеницы Саратовская 29 (С29) c густо опушенными листьями, несущий доминантные аллели генов Hl1 и Hl3, и две почти изогенные линии – i: С29 hl1, hl3 и i: С29 Hl2aesp с интрогрессией дополнительного гена опушения от диплоидного злака Aegilops speltoides. В контролируемых условиях климатической камеры изучены параметры флуоресценции хлорофилла, используемые для оценки физиологического состояния фотосинтетического аппарата растений, содержание фотосинтетических пигментов и активность ферментов аскорбат-глутатионового цикла в листьях С29 и линий. Устойчивость определяли с помощью комплексного индекса D, рассчитанного на основе изученных физиологических признаков. Рецессивное состояние генов опушения, как и введение дополнительного гена Hl2aesp, привели к 6-кратному снижению значений D. Под воздействием водного дефицита у линий менялся профиль параметров флуоресценции и снижался индекс жизнеспособности по сравнению с С29. Активность аскорбатпероксидазы, глутатионредуктазы и дегидроаскорбатредуктазы в листьях линии i: С29 hl1, hl3 уменьшалась в 1.9, 3.3 и 2.3 раза, в листьях линии i: С29 Hl2aesp – в 1.8, 3.6 и 1.8 раза соответственно по сравнению с С29. В условиях гидропонной теплицы изучена продуктивность линий. По сравнению с С29 у линии i: С29 hl1, hl3 при водном дефиците была снижена масса тысячи зерен. Продуктивность линии i: С29 Hl2aesp была значительно ниже независимо от условий водоснабжения в сравнении с С29. Предполагается, что выявленные эффекты связаны с нарушениями перекрестных регуляторных взаимодействий белков сети формирования трихом и факторов транскрипции, которые контролируют рост растений и реакцию на стресс

## Introduction

The spring bread wheat (Triticum aestivum L.) variety Saratovskaya 29 (S29) is one of the most famous varieties created in Russia, as it has high drought tolerance and outstanding grain quality (Ilyina, 1989). These properties characterize S29 as a valuable genetic resource, used for obtaining not less than 155 other varieties. One of the characteristic features of the variety is the dense pubescence of the leaf blade. Among 47 genotypes of bread wheat and relative species studied for the diversity of this trait, the leaf pubescence in S29 was distinguished by its high density and trichomes length (Pshenichnikova et al., 2017). Obviously, such morphological adaptations make a significant contribution to the drought tolerance of this variety. 

The trichomes are best known as excess sunlight reflectors (Ehleringer et al., 1976). The recent studies showed that trichomes can play a significant role in the water balance of leaves, affecting their wettability, droplet retention, and water absorption (Bickford, 2016). The dense trichomes layer can increase water use efficiency indirectly, promoting dew formation and reducing the difference in water potential inside the leaves and in the air. This allows stomata to be kept open longer, allowing for an influx of carbon dioxide without excessive water loss (Konrad et al., 2015). 

Among the cultivated plant species, the physiological role of leaf pubescence is poorly studied. In Oryza sativa L. introgression of a chromosome segment from the wild species Oryza nivara increased leaf pubescence, reduced transpiration rate and increased water use efficiency due to increased stability of the boundary air layer (Hamaoka et al., 2017). The only experiment in T. aestivum L. showed that the stomatal conductivity and the photosynthetic rate in substituted and near-isogenic lines with genetically different leaf pubescence were inversely proportional to the density and trichomes length (Pshenichnikova et al., 2019).

In bread wheat, several genes are known today that determine a different phenotype of leaf pubescence. The Hl1 and Hl2 genes were localized and mapped on chromosomes 4B and 7B, respectively (Maystrenko, 1976; Taketa et al., 2002; Dobrovolskaya et al., 2007). The Hl3 gene not yet assigned to a specific chromosome was genetically detected in the spring cultivar S29 (Doroshkov et al., 2011). In addition to them, the gene Hl2aesp allelic to the gene Hl2 was identified, introgressed into bread wheat from the species Ae. speltoides (Pshenichnikova et al., 2007). Hl1 and Hl3 affect to a greater extent on trichomes initiation and growth, while Hl2 regulates the length of trichomes (Doroshkov et al., 2016). Knowledge of the relationship of these genes with the physiological characteristics of drought tolerance and grain productivity is necessary for their including in the breeding process. 

Two near-isogenic lines with a different composition and allelic state of Hl genes were developed on the genetic base of the drought-tolerant wheat cultivar S29. The line i: S29 hl1, hl3 carry the recessive alleles of Hl1 and Hl3 genes which are dominant in the recipient. The line i: S29 Hl2aesp carries the gene for a long pubescence in addition to the two own dominant genes of the recipient. Previously, photosynthetic indicators were studied in these two lines under natural light and contrasting water supply. The lines were found to be contrast in terms of gas exchange (Pshenichnikova et al., 2019). However, no clear answer was obtained in respect of the pubescence influence on the parameters of chlorophyll fluorescence, which describe the physiological state of the plants photosynthetic apparatus (Goltsev et al., 2016).

The aim of this work was to assess the drought resistance of wheat by a wide range of physiological characteristics and productivity, depending on the presence of dominant or recessive alleles of the genes or the additional Hl2aesp gene, which determine the phenotype of leaf pubescence. Among the physiological traits were chlorophyll fluorescence indicators, including the OJIP-test parameters, the content of photosynthetic pigments and the effectiveness of the ascorbate-glutathione cycle, which, as know, is a powerful defense of cellular structures from oxidative damage (Foyer, Shigeoka, 2011). The resistance to drought was assessed with using the comprehensive score of drought D (Cao et al., 2015), calculated on the basis of the tolerance indexes of physiological traits.

## Materials and methods

Plant material. The object of the research was the droughttolerant wheat spring cultivar S29 carrying two genes (Hl1 and Hl3) for leaf pubescence and two near-isogenic lines with contrasting leaf pubescence. Line i: S29 hl1, hl3 was obtained by crossing the S29 cultivar with the non-pubescent Rodina cultivar carrying the recessive alleles of these genes. In the process of 8-fold backcrossing on the recipient cultivar, non-pubescent plants were selected. Line i: S29 Hl2aesp was obtained by crossing the S29 cultivar with the introgressed line 102/00i , which carries the Hl2aesp gene from the Ae. speltoides. Then, an 8-fold backcrossing was carried out on the recipient cultivar with the selection of plants bearing introgressed pubescence. The line i: S29 hl1, hl3 has a poor pubescence, while the leaves of the second line were densely pubescent. The origin, genetic characteristics and the quantitative characteristics of the pubescence in leaves of the near-isogenic lines have been described in detail earlier (Doroshkov et al., 2016; Pshenichnikova et al., 2019).

Experimental conditions. Physiological parameters were studied under controlled conditions of the climatic chamber CLF PlantMaster (CLF Plant Climatics GmbH, Wertingen, Germany), mounted in the phytotron of Siberian Institute of Plant Physiology and Biochemistry of Siberian Branch of the Russian Academy of Sciences (Irkutsk, Russia). The mixture consisted of humus, sand and peat (1:1:1) was used as soil for plant growing. A 16-hour photoperiod was maintained with a light intensity of 300 µmol (photon)/m–2 ·s–1, a day/night temperature 23/16 °C and a relative humidity of 60 %. Each pot (19 cm diameter, 0.24 cm high, containing 4 kg soil) was planted with ten grains. For each line, one pot was maintained in a state of optimal water supply, which was 60 % of the total soil moisture capacity (control), while in the second pot, starting from the stage of three leaves, watering was limited until the water content in the soil decreased to 30 % from the full moisture capacity of the soil (water shortage or drought). This model of drought corresponds to the climatic conditions of Western and Eastern Siberia in the spring.

Yield components of the lines was studied in a hydroponic greenhouse in the Institute of Cytology and Genetics of Siberian Branch of the Russian Academy of Sciences (Novosibirsk, Russia) during two seasons. Plants were grown in the bathtubs (size: 4×1×0.35 m) filled with artificial soil “ceramzit” (expanded clay), Knop’s solution was used for plant nutrition. The near-isogenic lines and S29 were grown in rows in two independent replicates consisted of seven plants. From seedlings to tillering stages, all plants in the bathtubs were watered twice a day. After the beginning of tillering, two water supply regimes were created in the bathtubs. At a control regime, plants were continued to water twice a day until the end of a season. In the second regime, water supply was stopped. Moisture level was measured once a week on the depth 6 cm using a moisture meter MG-44 (“AKVASENSOR”, Kharkov, Ukraine). The moisture value in the control variant was 28–30 % in average during the season. In the second variant, the moisture level gradually decreased from the control level and after a month of drought reached the constant value of 10–12 % in average. The following yield components were measured: number of tillers, stem and spike length, the number and weight of grains in main and secondary spikes. Thousand grain weight was a calculated value.

**Fig. 1.**
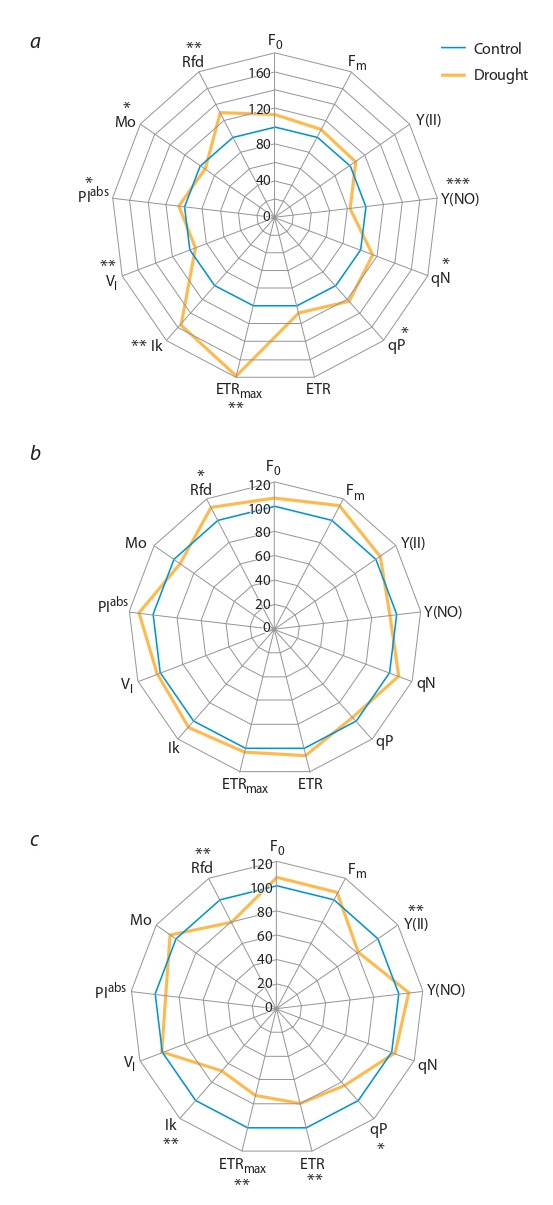
Relative deviation of chlorophyll fluorescence parameters (in %) under drought compared to control (watering 100%) in S29 (a) and lines i: S29 Hl2aesp (b) and i: S29 hl1, hl3 (c). * p < 0.05; ** p < 0.01; *** p < 0.001. F0 – minimal fluorescence yield of the dark-adapted state; Fm – maximal fluorescence yield of the dark-adapted state; Y(II) – real quantum yield of PSII; Y(NO) – quantum yield of unregulated fluorescence quenching; qN – coefficient of non-photochemical fluorescence quenching; qP –coefficient of photochemical fluorescence quenching; ETR – rate of electron transport provided by PSII; ETRmax – maximum electron transport rate; lk – intensity of illumination, expressing the beginning of PAR saturation; VI – relative variable fluorescence at 30 ms; Мо relative the closing rate of the reaction centers of PSII; PIabs – PSII performance index; Rfd – PSII vitality index

Determination of Chl fluorescence parameters. The measurements of the Chl fluorescence of leaves were carried out using a portable impulse fluorometer PAM-2500 (Walz, Effelrich, Germany). A total of 33 Chl fluorescence parameters were measured and calculated. 13 of them were most sensitive to water scarcity, and are shown in Fig. 1. In order to register the minimal fluorescence yield of the dark-adapted state (F0), we darkened the leaves for 30 min and then illuminated them with modulated measuring light of low frequency (5 Hz) and low intensity (630 nm). The intensity of the Chl fluorescence under conditions of closed reactive centers (Fm) was measured after the exposure of a light impulse of high intensity (25,000 µmol (photon)/m–2 ·s–1, 630 nm). In addition, we calculated the rate of electron transport (ETR), the real quantum yield of PSII (Y(II)), quantum yield of unregulated fluorescence quenching (Y(NO)), coefficient of non-photochemical fluorescence quenching (qN), coefficient of photochemical fluorescence quenching (qP). Parameters lk and ETRmax were calculated from the Chl fluorescence light curve (PAR range from 0 to 2,000 µmol (photon)/m–2 ·s–1)

The quantitative analysis of the characteristics of photosynthesis primary processes based on parameters of fluorescence kinetic curve was conducted using the OJIP-test, based on the theory of energy pathways (Strasser et al., 2004). The following parameters were calculated:

- VI = (F30ms – F0)/Fv – relative variable fluorescence at 30 ms;
- PIabs = (RC/ABS) × [φPo /(1 – φPo)] × [Ψ0 /(1 – Ψ0)] – performance index, an indicator of the functional activity of PSII;
- Мо = 4×(F0.3ms –F0)/(Fm –F0) – the parameter reflects the closing speed of the reaction centers of PSII;
- Rfd = (Fm –Ft)/Ft – viability index (Lichtenthaler et al., 2005).

Determination of photosynthetic pigments content and enzymes activity in leaves. After determining the photosynthetic parameters, the leaf pieces were frozen with liquid nitrogen and stored at the temperature of –80 °C. The content of pigments per gram of leaves dry mass and activities of superoxide dismutase (SOD), glutathione reductase (GR), dehydroascorbate reductase (DHAR) and ascorbate peroxidase (APX) were determined and calculated as it was previously described (Osipova et al., 2016). 

Statistical analysis. Chl fluorescence was measured on the flag leaves of four plants per line. The content of pigments and the enzymes activity were determined in three biological and three analytical replicates. One plant of each line was taken for the biological replicate. Yield components were studied in each season, in two replicates; in all, the measurements were made for twenty-four plants of each line under drought and in control conditions. All the comparisons were made with S29. Microsoft Excel 2010 (Microsoft Corp., Redmond, WA, USA) was used for data processing and histogram plotting. The statistical significance of the differences between the recipient variety and the wheat lines from the measured parameters was compared with the Student’s test. Means were considered to be significantly different when p < 0.05. The statistics package PAST (Hammer et al., 2001) was used for principal component analysis (PCA). The drought tolerance index (IT, %) for each parameter was calculated as shown in the following formula (1):

**Formula:**
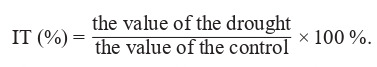
(1)

The data from PCA were used in further calculations of comprehensive drought tolerance values D (Cao et al., 2015).

## Results

Effect of water deficit on fluorescence parameters of S29 and lines i: S29 Hl2aesp and i: S29 hl1, hl3. The reaction of the photosynthetic apparatus to water deficiency was significantly different in the studied wheat genotypes. The thirteen parameters most sensitive to water deficit are shown in Fig. 1.

In S29, the most noticeable changes were an increase of ETRmax and lk, statistically significant increase in qP, qN, PIabs and vitality index Rfd. Parameters Y(NO), Mo, and VI in variety S29 decreased under conditions of water deficiency (see Fig. 1, a). In the i: S29 Hl2aesp line the chlorophyll fluorescence parameters remained unchanged under water deficiency, with the exception of an increase in the viability index Rfd (see Fig. 1, b). In line i: S29 hl1, hl3, under water deficiency, the parameters Y(II), qP, ETR, ETRmax, lk, and Rfd decreased statistically significantly compared to the control (see Fig. 1, c).

**Fig. 2.**
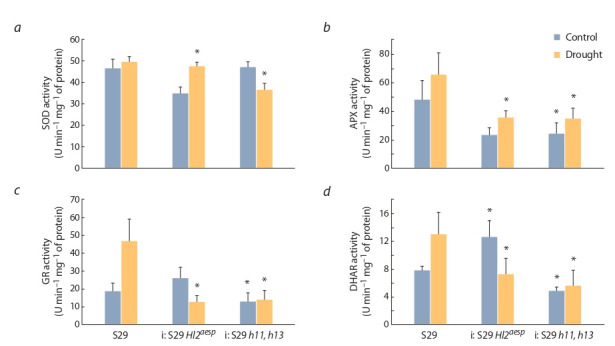
The average activity of superoxide dismutase (SOD), ascorbate peroxidase (APX), glutathione reductase (GR) and dehydroascorbate reductase (DHAR) in the leaves of S29 and lines i: S29 Hl2aesp and i: S29 hl1, hl3 under normal irrigation and drought. * Significant differences with S29, p < 0.05.

Effect of water deficit on the antioxidant enzymes activities in leaves of S29 and lines i: S29 Hl2aesp and i: S29 hl1, hl3. Under adaptation to water deficiency of cv. S29, the activity of APX, GR, and DHAR in leaves were higher than under optimal conditions (Fig. 2, b–d ).

In the leaves of line i: S29 Hl2aesp the activities of this enzymes were significantly lower compared to S29 under drought conditions; moreover, GR and DHAR activities in this line were lower during drought compared to the control. The activities of APX, GR and DHAR in the line i: S29 hl1, hl3 were significantly reduced compared to S29 regardless of water supply conditions. SOD activity was also reduced compared to S29 under drought conditions in lines, most significantly in the line i: S29 hl1, hl3 (see Fig. 2, a).

Effects of water deficit stress on the photosynthetic pigments content in leaves of S29 and lines i: S29 Hl2aesp and i: S29 hl1, hl3. The content of chlorophylls and carotenoids in the leaves of S29 did not change depending on the water supply conditions (Suppl. Material 1)1. Under optimal irrigation conditions, the lines significantly exceeded the initial variety in the content of photosynthetic pigments. Under conditions of water deficiency, the content of chlorophylls and carotenoids in the lines decreased. In the line i: S29 Hl2aesp, the decrease in the content of chlorophyll b and carotenoids was significantly lower than in S29. Regardless of the conditions, the ratio chlorophyll a+b / carotenoids was higher in the line i: S29 hl1, hl3 compared to the original variety. This is due to the higher content of chlorophylls in the leaves of this line. The tolerance index of photosynthetic pigment content in both lines was reduced compared to S29 (Suppl. Material 2).

Supplementary Materials 1–3 are available in the online version of the paper: http://vavilov.elpub.ru/jour/manager/files/SupplOsipova_engl.pdf


Principal component analysis and calculation of the comprehensive evaluation value. The drought tolerance coefficients for 14 physiological traits were involved into PCA (see Suppl. 2). The cumulative contribution rates of PC1 and PC2 accounted for 100 % of the total variation (Suppl. Material 3). PC1 accounted for 84.9 % of total variation and was constituted mainly by ITs of GR and DHAR activities and ETRmax. PC2 explained 15.1 % of the total variation with SOD and DHAR activities, ETRmax and lk being the largest contributors. Using the formulas of X. Cao et al. (2015), the comprehensive evaluation value, D, was calculated. The D value indicated the relative level of drought tolerance in the different wheat genotypes subjected to drought stress. Based on this criterion, S29 with a D value of 0.948 had the highest drought tolerance. The lines i: S29 Hl2aesp and i: S29 hl1, hl3 had D value of 0.150 and 0.160, respectively. Thus, a comprehensive value D, which takes into account 14 physiological indicators, showed that both near-isogenic lines had a significantly reduced level of resistance compared to the original cultivar. The greatest contribution to these differences was made by such indicators as the activity of GR and DHAR, as well as the parameters of the light curve ETRmax and lk.

The productivity evaluation of S29 and lines i: S29 Hl2aesp and i: S29 hl1, hl3 in different conditions of water supply. The recipient variety significantly exceeded the line with the additional gene Hl2aesp for leaf pubescence. Most of the yield components of the line i: S29 Hl2aesp were significantly reduced, regardless of the water supply conditions (Table).

**Table 1.**
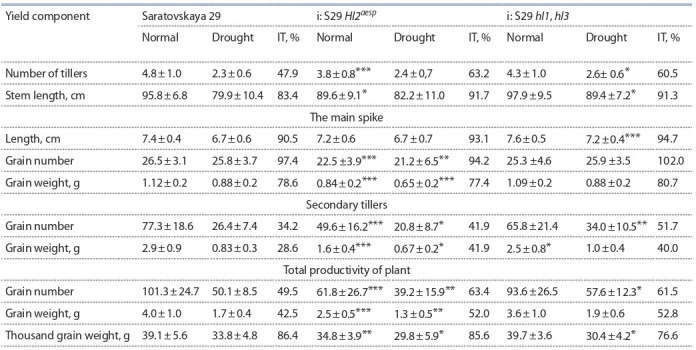
Average values of yield components in cv. Saratovskaya 29 and the lines i: S29 Hl2aesp and i: S29 hl1, hl3 under normal watering and drought grown under hydroponic green-house conditions on artificial soil * p < 0.05; ** p < 0.01; *** p < 0.001 in comparison with S29 on corresponding watering regime

Inhibition of plant development was observed starting from tillering; a reduced yield was formed both on the primary and secondary spikes. This line was also significantly inferior in productivity to the line with recessive genes Hl1 and Hl3 for leaf pubescence. Cv. S29 reduced the productivity of secondary tillers under drought, but thousand grain weight decreased slightly. The line i: S29 hl1, hl3 differed from S29 in reduced productivity of the secondary tillers under irrigation conditions (see Table). Under drought, it exceeded the recipient in length of the stem and the main spike, the number of grains in the secondary spikes and the total number of grains. However, thousand grain weight was lower compared to the original cultivar. That is, the line i: S29 hl1, hl3 formed smaller grains under water deficiency

## Discussion

Dense pubescence of the leaf blade is a morphological component of adaptation of cv. S29 to drought conditions (Ilyina, 1989; Pshenichnikova et al., 2017, 2019). On its basis, the two near-isogenic lines with genetically modified morphology of leaf pubescence were obtained. The line i: S29 hl1, hl3 with recessive genes for this trait is characterized by a significant decrease in the density (6.6–14 times) and length (2.5–4.7 times) of trichomes on different sides of the leaf and under different conditions compared with S29. In line i: S29 Hl2aesp with gene introgression from Ae. speltoides, the density of trichomes on different sides of the leaf and under different conditions increased 1.08–1.17 times, and the length of trichomes increased 1.6 times compared to the recipient (Doroshkov et al., 2016; Pshenichnikova et al., 2019). The lines are a convenient model for studying the role that pubescence genes play in wheat stress tolerance. Previously, we used the lines to assess the relationship between the density and trichomes length and gas exchange parameters (Pshenichnikova et al., 2019). In a greenhouse with natural light, the transpiration rate, stomatal conductivity, and the rate of photosynthesis of S29 and lines were inversely proportional to the density and length of trichomes, which is consistent with the data of N. Hamaoka et al. (2017) for rice. The highest water use efficiency, calculated as the relation photosynthesis rate / transpiration rate was in S29. The water use efficiency at line i: S29 hl1, hl3 was 1.9 times lower under optimal conditions and 1.5 times lower under drought compared to S29, since increased transpiration led to water loss (Pshenichnikova et al., 2019). 

An analysis of the chlorophyll fluorescence parameters in this experiment showed that, when adapting to drought, S29 was characterized by a significant increase in the light curve parameters ETRmax and lk, an increase in the qP parameter, and a significant (30%) increase in the PSII viability coefficient Rfd. These data indicate that S29 can stably support PSII functions, increasing the fraction of light energy used for photochemical reactions and the rate of assimilation of photosynthetic CO2 under drought conditions (Lichtenthaler et al., 2005). The content of photosynthetic pigments was also stable. A significant increase in APX, DHAR, and GR activity was observed in S29 leaves under drought which contributed to the maintenance of structural and functional the integrity of the photosynthetic apparatus and the maintenance of the ascorbic acid (Asc) pool (Foyer, Shigeoka, 2011). Under conditions of water deficiency Asc, in addition to the antioxidant role, can be the donor of electrons in the photosynthetic electron transport chain (Tóth et al., 2013). Thus, at the cellular level, the high drought tolerance of S29 was associated with a high antioxidant ability and preservation of the functions of the photosynthetic apparatus (PhA).

Introduction of the additional pubescence gene Hl2aesp into the genotype of S29 led to an increase in the length of trichomes (Pshenichnikova et al., 2019) and significant changes in the physiological responses to water deficiency. Unlike S29, the chlorophyll fluorescence parameters of the line i: S29 Hl2aesp did not change under drought compared to optimal conditions except for a slight increase in the Rfd index. At the same time, APX, GR, and DHAR activities in the line were reduced 1.8, 3.6, and 1.8 times, respectively, compared with the recipient. Since maintaining the redox state of Asc through recycling is critical under stressful conditions (Gallie, 2013), a significant decrease in DHAR activity in the line i: S29 Hl2aesp could lead to a decrease in the Asc content. At low concentrations of Asc, the activity of APX into chloroplasts is rapidly lost in the presence of H2O2. These in turn limits the effectiveness of photosynthesis under stressful conditions (Ishikawa, Shigeoka, 2008). The high content of chlorophylls and carotenoids comparable to the recipient S29 under optimal conditions did not retained under drought. It is likely that a significant decrease in the line productivity, both under favorable conditions and under drought, is associated with the observed inhibition of physiological processes. 

The recessive state of the Hl1 and Hl3 genes in the line i: S29 hl1, hl3 also led to a significant weakening of the antioxidant potential. As in the previous line, SOD, APX, GR, and DHAR activities under drought were reduced at the same manner: 1.4, 1.9, 3.3, and 2.3 times, respectively, compared with the recipient S29. Chlorophyll fluorescence parameters indicated disturbances in the functioning of PhA under stress, since the real efficacy of PSII, ETR and ETRmax, PSII viability coefficient (Rfd), and photosynthetic fluorescence quenching (qP) significantly decreased. The content of leaf pigments also decreased. Trichomes formation and accumulation of phenolic compounds are interconnected at the molecular level (Pattanaik et al., 2014; Zhang, Schrader, 2017). Due to the diffuse deposition of phenolic compounds in the cell walls, trichomes provide a protection against ultraviolet radiation by acting as optical filters, shielding wavelengths that can damage sensitive tissues (Karabourniotis et al., 2020). Therefore a further increase of a light load may lead to even more dramatic changes in the operation of the photosynthetic apparatus of the i: S29 hl1, hl3 line. Changes in yield components of the line i: S29 hl1, hl3 were less pronounced compared to the line i: S29 Hl2aesp. Under drought, it was even more productive than the recipient cultivar. The increase in productivity was due to the number of grains of the secondary spikes. However, the line gave smaller grains which negatively affects the output of flour. It can be assumed that the decrease in the stability of the photosynthetic apparatus found in the line led to a disruption in the synthesis of simple carbohydrates in line i: S29 hl1, hl3. This, in turn, reduced the level of starch synthesis associated with 1,000 grain mass and productivity (Wang et al., 2019). The values of the comprehensive drought tolerance index D, calculated on the basis of physiological parameters, in the lines were 6 times lower compared to S29

The genetic regulation of trichomes formation in wheat has not been studied enough to unambiguously explain the reasons for the negative impact of manipulations with the Hl1, Hl3, and Hl2aesp genes on tolerance to water deficiency. A well-studied genetic network for the development and differentiation of Arabidopsis trichomes may be a model in this regard. Dozens of genes are involved in this network. The vast majority of the products of these genes are transcription factors. They are components of the regulatory network of trichomes initiation, root hairs formation, and flavonoid biosynthesis involved in a large number of cross-regulatory protein-protein interactions (Pesch, Hülskamp, 2004, 2009; Pattanaik et al., 2014; Zhang, Schrader, 2017). For example, P. Achard et al. (2008) showed that transcription levels of Cu/Zn superoxide dismutase are positively modulated by proteins of the DELLA regulatory protein family. However, DELLA proteins interact with the WD-repeat/bHLH/MYB complex, which is involved in the regulation of development of trichomes (Qi et al., 2014). The transcription factors GIS and GIS2 play an important role in the integration of cytokinin and gibberellin signaling and have regulatory interactions with the proteins of the trichomes initiation network GL1, SRY and GL3, thereby affecting the functioning of the initiating complex of trichomes formation (Gan et al., 2007). These and other examples available in the literature indicate that genes that control the development of trichomes are linked by cross-regulatory interactions with transcription factors that regulate hormonal signaling, stress responses, including antioxidant response and developmental programs. Based on knowledge of the regulation of trichomes formation in Arabidopsis, we assume that the effects identified in our work, namely, the negative impact on physiological stability and yield of wheat of the recessive state of the Hl1 and Hl3 genes or the introgression of the Hl2aesp gene, are probably associated with violations of cross-regulatory protein-protein interactions prevailing in the genotype of the recipient cultivar S29.

## Conclusion

Changes in the composition and allelic state of Hl genes influenced not only the quantitative characteristics of leaf pubescence, but also stability of photosynthetic pigments content, chlorophyll fluorescence indexes, activity of ascorbateglutathione cycle enzymes, and productivity of near-isogenic lines of bread wheat. The comprehensive drought tolerance index D, calculated on the basis of physiological indicators, was 6 times lower in the lines compared to S29. Regardless of the water supply conditions, all yield components significantly decreased in the line i: S29 Hl2aesp, and in the line i: S29 hl1, hl3 the weight of 1,000 grains decreased as compared to S29. It is assumed that these effects are associated with changes in the cross regulatory interactions of proteins involved in the formation of trichomes, and transcription factors that regulate growth, development, and reactions to stress factors.

## Conflict of interest

The authors declare no conflict of interest.
